# Nurturing Infants to Prevent Atopic Dermatitis and Food Allergies: A Longitudinal Study

**DOI:** 10.3390/nu16010021

**Published:** 2023-12-20

**Authors:** Emilia Vassilopoulou, Dimitrios Rallis, Gregorio Paolo Milani, Carlo Agostoni, Gavriela Feketea, Maria Lithoxopoulou, Evangelia Stefanaki, Fani Ladomenou, Nikolaos Douladiris, Caoimhe Cronin, Codruta Alina Popescu, Raluca Maria Pop, Ioana Corina Bocsan, Sophia Tsabouri

**Affiliations:** 1 Pediatric Unit, Fondazione IRCCS Ca’ Granda Ospedale Maggiore Policlinico, 20122 Milan, Italy; milani.gregoriop@gmail.com (G.P.M.); carlo.agostoni@unimi.it (C.A.); 2 Neonatal Intensive Care Unit, Faculty of Medicine, University of Ioannina, 45500 Ioannina, Greece; drallis@uoi.gr (D.R.); stsabouri@gmail.com (S.T.); 3 Department of Clinical Sciences and Community Health, Università degli Studi di Milano, 20122 Milan, Italy; 4 Department of Pharmacology, Toxicology and Clinical Pharmacology, University of Medicine and Pharmacy, 400337 Cluj-Napoca, Romania; gabychri@otenet.gr (G.F.); raluca.pop@umfcluj.ro (R.M.P.); bocsan.corina@umfcluj.ro (I.C.B.); 5 Pediatric Allergy Outpatient Clinic, Department of Pediatrics, “Karamandaneio” Children’s Hospital of Patra, 26331 Patras, Greece; 6 2nd Department of Neonatology, School of Medicine, Faculty of Health Sciences, Aristotle University of Thessaloniki, “Papageorgiou” General Hospital of Thessaloniki, 56403 Thessaloniki, Greece; mlithoxopoulou@yahoo.com; 7 Pediatric Allergy Outpatient Clinic, Department of Pediatrics, Venizeleion General Hospital of Heraklion, 71409 Heraklion, Greece; linastef74@gmail.com; 8 Pediatric Infectious Unit, Department of Pediatrics, University Hospital of Ioannina, 45500 Ioannina, Greece; fladomenou@gmail.com; 9 Allergy Unit, 2nd Pediatric Clinic, University of Athens, 11527 Athens, Greece; ndouladiris@gmail.com; 10 Department of Paediatrics and Child Health, University College Cork, T12 K8AF Cork, Ireland; caoimhecronin@ucc.ie; 11 Department of Abilities Human Sciences, Iuliu Hatieganu University of Medicine and Pharmacy, 40012 Cluj-Napoca, Romania; cpopescu@umfcluj.ro

**Keywords:** atopic dermatitis, diet diversity, food allergy, atopic march, siblings, mode of birth, prenatal smoking

## Abstract

Background: Atopic dermatitis (AD) at a young age often precedes the development of food allergies. Although AD affects millions of infants worldwide, prenatal and postnatal risk factors, and their association with the development of food allergies later on, are not fully elucidated. This study seeks to investigate AD epidemiology in infancy and its risk factors, examining early-life factors (both prenatal and postnatal) that could contribute to the later development of food allergies. Methods: Between January 2019 and December 2019, 501 infants were included in this prospective cohort study. Longitudinal data collection was performed through maternal interviews, the first one conducted within three days after the delivery and the second within 24 to 36 months after the delivery, encompassing variables such as demographics, family history of atopy, maternal smoking, antibiotic use during pregnancy, the mode of delivery, breastfeeding history, food practices, and greenness exposure within 3 days from delivery, while they were still in the hospital. Results: Maternal smoking during pregnancy (*p* = 0.001) and an older sibling atopy history (*p* = 0.03) was significantly linked to AD incidence. Cesarean section delivery (*p* = 0.04) was associated with a higher risk of food allergies in infants with AD. Having a garden at home correlated with a higher likelihood of AD (*p* = 0.01), and food elimination without medical guidance (*p* = 0.02) due to AD correlated with an elevated risk of food allergies. Conclusions: Encouraging timely allergenic food introduction while promoting dietary diversity, rich in plant-based foods, maternal smoking cessation, and professional dietary guidance may help minimize AD and food allergy risk. Future studies should address the role of greenness in the development of AD and food allergies.

## 1. Introduction

Atopic dermatitis (AD), commonly known as eczema, is a chronic or chronically relapsing inflammatory skin condition primarily manifesting in childhood, with some severe cases persisting into adulthood [1]. It ranks among the most prevalent noncommunicable skin diseases globally, affecting up to 20% of children and 2–8% of adults [1]. In Greece, AD holds the top position among diagnosed skin conditions in children, accounting for 20.7% of pediatric dermatology referrals, highlighting its high prevalence in the region [2].

The pathophysiology of AD is intricate and multifaceted, arising from the interplay of environmental factors, compromised skin barriers, variations in the skin microbiome, and immune dysregulation among individuals genetically predisposed to AD [3]. Among modifiable risk factors, passive smoking stands out as a significant contributor to AD development [4].

Early-life dietary practices have been recognized as crucial for AD prevention for decades. Recommendations dating back to the 1930s endorsed exclusive breastfeeding for at least three months as a protective measure against AD [5]. However, this advice was debated in the early 2000s, with studies, including a Cochrane review, finding no added benefit of exclusive breastfeeding for six months compared to exclusive breastfeeding for three to four months followed by mixed breastfeeding (introduction of complementary liquid or solid foods while continuing breastfeeding) until six months in preventing AD [6]. More recently, a large-case control study in 2016 suggested that early weaning, defined as introducing solid foods at 4 or 5 months of age, reduces the risk of AD. Children weaned at 4 months showed a lower risk of AD compared to those exclusively breastfed, with the introduction of a variety of solid foods further diminishing the risk of AD [5]. This highlights the potential protective effect of introducing a diversified diet from four months of age while gradually incorporating solid foods alongside breastfeeding [5].

AD serves as the initial manifestation in the natural progression of allergic diseases, a phenomenon commonly referred to as the atopic or allergic march [7]. Frequently, dry skin observed at birth advances to AD within the first 2–3 months of life, acting as a precursor to subsequent atopic conditions, including food allergies, allergic rhino-conjunctivitis, and asthma [8]. In fact, food allergies to common items like hen’s eggs and cow’s milk can exacerbate AD in 30% of affected children [1]. Dietary elimination of these allergens is often recommended to alleviate AD symptoms. However, it is worth noting that elimination diets can negatively impact quality of life and increase the risk of future anaphylactic reactions [1].

Current discussions are focused on identifying primary prevention methods for AD and mitigating the development of additional atopic conditions, particularly food allergies in individuals with AD [9,10]. Furthermore, there is a growing need to adopt a multifaceted approach in identifying prenatal and dietary risk factors for AD, as well as factors influencing the development of other atopic conditions in individuals with AD [11]. Consequently, this study aims to explore prenatal and postnatal risk factors for AD development and identify individuals at heightened risk of developing other atopic conditions, specifically food allergy, through a prospective cohort study.

## 2. Methods

### 2.1. Study Population

The study recruited pairs of mothers and infants born in the Thessaloniki and Crete regions between January 2019 and December 2019. Mothers with a diagnosis of a chronic disease that used medication for its treatment, such as diabetes, autoimmune disease, cardiovascular disease, gestational diabetes, and/or infants born with a birth defect (congenital anomaly), were excluded from the study.

Ethical approval for the study was obtained from the Venizeleio hospital Ethical Committee (ref. no. 08/130618_50(2)), and written informed consent was obtained from each participating mother, in accordance with the principles outlined in the Declaration of Helsinki.

### 2.2. Study Design and Data Collection

After enrolment, mothers were invited to answer structured questionnaires through interviews. The initial round of interviews with women took place within 3 days from delivery, while they were still in the hospital. These interviews were performed in person by a trained researcher with an expertise in allergy (pediatric allergist or dietitian with an allergy specialty). A subsequent telephone interview was conducted within 24 to 36 months after the delivery.

During the first interview, the following data points about the newborn were collected: demographic data, including sex, place of birth, nationality, and parental education levels, parental history of atopy and any atopic history in older siblings, neonatal gestational age, birth weight, mode of delivery, parental smoking during pregnancy, and antibiotic use during pregnancy.

During the second interview, the following information about the infant was gathered: the current age of the infant, any doctor-diagnosed food allergy, history of asthma/viral-induced wheeze, and food anaphylaxis. Information regarding the breastfeeding (including history and duration of exclusive breastfeeding), parental smoking during the breastfeeding period, and antibiotic use after birth, the presence of younger siblings and any atopy history in them, the presence of a garden at home, and ownership of pets were also noted. Moreover, information regarding the complementary feeding practices were explored, including the time of initiation, the timing of the introduction of each food category (fruits, vegetables, meat, dairy, starchy products, fats, and lipids), and the first five foods introduced from each food category were recorded. Foods not introduced until the first year, and the reasons for the non- or late introduction, were noted. Data on the presence of emergency kits for the management of food allergic reactions, in the case of food allergy diagnosis and physician-recommended food elimination, and food-elimination practices without the physician’s guidance, were gathered, together with the reasons behind these eliminations and the specific types of foods that were removed from the study population’s diet. Finally, participant stratification was determined by the confirmed medical diagnosis of AD conducted by a pediatric allergist, with a notation of the age at which the first diagnosis occurred.

### 2.3. Statistical Analysis

Continuous variables were expressed as mean ± standard deviation or median (interquartile range), whereas the categorical variables were expressed as n (percentage %). The normality of the distributions of the continuous variables was assessed by the Kolmogorov–Smirnov. Comparisons between continuous variables were performed with the Student’s *t*-test or Mann–Whitney test, whereas comparisons between the categorical variables utilized the chi-square test or the Fisher’s exact test, as appropriate.

Multivariate logistic regression was used to determine variables associated with AD (dependent variable), including those independent variables with a significant effect on the univariate analysis; namely, the place of living, the mode of delivery, living in a house with a garden, maternal smoking in pregnancy, paternal atopy history, and atopy history of older siblings. In addition, multivariate logistic regression was used to determine variables associated with food allergy in children with atopic dermatitis AD (dependent variable), including independent variables with a significant effect on the univariate analysis; namely, unsupervised food elimination before the onset of food allergy and the mode of delivery. Odds ratios (OR) and 95% confidence intervals (CIs) were calculated. All performed tests were two-sided and a *p*-value less than 0.05 was considered statistically significant (alpha 0.05). Missing data were managed with standard listwise deletion. The data were analyzed using SPSS Statistics (IBM SPSS Statistics for Windows, Version 24.0. Armonk, NY, USA).

## 3. Results

Among the 519 pairs of mothers and infants initially recruited, 501 completed the follow-up study, comprising 87 infants with an AD diagnosis and 414 without AD. The sex distribution was similar between the AD and non-AD groups (56% vs. 53%, *p* = 0.64).

The majority (92%) held Greek nationality, with no significant variation between AD and non-AD groups (93% vs. 94%, *p* = 0.80).

The age at AD diagnosis ranged from 2 to 7 months (median: 5.5 months). Food allergies exhibited a significant disparity between AD and non-AD groups (*p* = 0.008).

Physician-recommended food elimination by doctors aiming to reduce AD exacerbations was recorded in 10% of AD infants. Additionally, unsupervised food elimination showed a significant difference between the two groups (37.9% AD vs. 2.9% non-AD, *p* = 0.004), with the main reasons for food elimination being the high-allergenic potential of the food as considered by the mother and the child’s refusal to try new foods (Table 1).

### 3.1. Familial Factors

Maternal and paternal education levels demonstrated no significant discrepancies between AD and non-AD groups. However, the maternal and paternal atopy histories exhibited significant associations with AD (24.13% AD vs. 4.98% non-AD, *p* = 0.049 and 27% AD vs. 21.98% non-AD, *p* = 0.02, respectively).

Maternal and paternal smoking during pregnancy and breastfeeding featured significant associations with AD incidence, with a higher prevalence in the AD group (Table 2). Maternal antibiotic use during pregnancy did not differ significantly between the two groups. The presence of older siblings significantly differed between the AD and non-AD groups (66.66% AD vs. 82.12% non-AD, *p* = 0.02). Moreover, older sibling atopy history also displayed a significant difference, with a higher prevalence in the non-AD group (22.41% AD vs. 7% non-AD, *p* = 0.005).

### 3.2. Perinatal and Early-Life Characteristics

The mean gestational age hovered at 34.4 weeks, with no significant difference between AD and non-AD groups. Similarly, birth weight showed no significant difference. The mode of delivery exhibited a notable difference between AD and non-AD groups, with cesarean section being more frequent among non-AD infants (*p* = 0.006).

The duration of breastfeeding and exclusive breastfeeding displayed no significant variations between AD and non-AD groups. A notable difference on food elimination was observed in breastfeeding, when mothers of AD infants more often eliminated milk, tree-nuts, peanuts, and pulses (including lentils, chickpeas, and beans) (*p* < 0.001).

A larger percentage of AD infants resided in houses with gardens (51% vs. 35%, *p* = 0.005). Approximately 32% of infants had pets, predominantly dogs (81%) (Table 3).

### 3.3. Complementary Feeding and Early Life Environmental Factors

No significant disparities were observed in the initiation of formula feeding or complementary feeding between AD and non-AD infants. AD infants experienced delays in introducing specific vegetables (mushrooms, green beans, okra, carrot, broccoli, sweet potato, onion, squash, celery, cabbage, peas, and cucumber) (*p* = 0.002) and fruits (strawberry, melon, watermelon, cherries, orange, lemon, pomegranate, and kiwi) (*p* = 0.02), but nuts were introduced earlier (*p* = 0.016) compared to non-AD infants. The type of fish introduced did not diverge significantly between AD and non-AD infants. Antibiotic use during infancy also did not exhibit a significant difference (Table 4).

### 3.4. Risk of AD

In Table 5, the univariate analysis shows that several factors are associated with an increased risk of AD in infants, including living in Crete, vaginal delivery (VD), having a house with a garden, maternal smoking during pregnancy, and paternal and older sibling atopy history. In the multivariate analysis, factors such as vaginal delivery (OR 3.11, 95%CI 1.27–7.61, *p* = 0.006), having a house with a garden (OR 3.84, 95%CI 1.50–9.80, *p* = 0.01), maternal smoking during pregnancy (OR 7.18, 95%CI 2.27–22.64, *p* = 0.001), and older sibling atopy history (OR 7.87, 95%CI 1.25–50.00, *p* = 0.03) remain significantly associated with a higher risk of AD.

### 3.5. Risk of Food Allergy in Infants with AD

Table 6 is dedicated to infants with AD and their susceptibility to food allergies. In both the univariate and multivariate analyses, factors such as unsupervised food elimination before the onset of food allergy (OR 6.63, 95%CI 1.31–33.46, *p* = 0.02) and cesarean section delivery (OR 3.58, 95%CI 1.05–12.19, *p* = 0.04) continue to show a significant association with an increased risk of food allergies.

## 4. Discussion

This prospective cohort study aimed to explore a multitude of factors associated with the incidence of AD in infants, shedding light on the complex interplay of heredity, demographics, environmental factors, and medical interventions during early life and the factors that might increase the risk of food allergy in this population. Our study supports that unsupervised food elimination in infants with AD reduces diet diversity and raises the food allergy risk.

Many caregivers unreasonably eliminated a wide range of foods, including major allergens, such as peanut, sesame, egg, milk, and shellfish, but also various fruits and pulses, without physicians’ guidance. It is noteworthy, however, that only in approximately 15% of the cases was elimination attributed to infants with AD refusing to eat the food, potentially indicating mild symptoms before a food allergy diagnosis. The remaining 28% who pursued an elimination diet without physicians’ guidance did so for other reasons. These included maternal fear, perceptions regarding allergenic or healthy foods, or concerns about exacerbating the AD. Past recommendations supported the delayed introduction of potentially allergenic foods in high-risk allergy infants, i.e., infants with AD or familial allergy history [12,13,14]. However, results from trials on high-risk allergy infants recommend introducing these foods between four to six months of age [15], and maintaining regular exposure to them to promote tolerance [16,17]. Previous research indicates that the late introduction or removal of such foods increases the risk of losing immune tolerance and experiencing food allergic reactions, and potentially hindering proper growth [18,19,20,21]. These findings stress the importance of the early introduction and consistent exposure to food allergens for preventing allergies in high-risk infants, which differs from our observations.

Furthermore, our study population exhibited the delayed introduction of foods, particularly fruits and pulses. This delay contributed to a decrease in dietary diversity. Fruits and pulses are known for their high dietary fiber and antioxidant content, which can have a notable anti-inflammatory impact, promoting immune tolerance [22]. These foods enhance the diversity and maturation of gut microbiota by acting as prebiotics and providing nourishment to tolerogenic microbes [23]. Hence, it is crucial to thoughtfully plan dietary strategies for AD management to prevent an elevated risk of other allergic conditions, particularly food allergies [24].

We additionally observed a noteworthy connection between the presence of AD in infants and an older sibling with a history of atopy. This emphasizes the hereditary aspect of this condition and highlights the significance of factoring in genetic predisposition when assessing an infant’s likelihood of developing AD [25].

Moreover, maternal smoking during pregnancy as a possible factor was linked to the occurrence of AD. This discovery is consistent with prior studies [26] that underscore the importance of both prenatal and postnatal smoking cessation [27,28]. Smoking has previously been recognized as a contributing factor to DNA methylation when offspring are exposed to it before birth [29,30,31]. As a result, a hypothesis suggests in-utero programming in early-life allergy development, which has been recently supported by a meta-analysis [32], which suggests smoking cessation for pregnant women and emphasizes the importance of avoiding secondhand smoke to prevent the development of AD [3].

Mode of delivery, stood out as a significant factor, with infants delivered vaginally showing a higher odds ratio for AD. Previous research supports that uncomplicated vaginal delivery is protective against allergic diseases, but research is limited for AD [33,34,35]. In their study, Mubanga et al., in 1,399,406 children, reported a higher risk of AD when children are delivered by instrumental vaginal delivery, emergency cesarean section, or elective cesarean section [33]. We assume that the result in our study is pertinent, especially given the notably high rate of cesarean sections within the specific population. Conversely, cesarean section was found to be associated with an increased risk of food allergies in infants with AD, in line with previous studies that have reported how cesarean section can disrupt gut bacterial colonization and lead to a reduction in specific beneficial bacteria [36].The presence of a home garden emerged as a significant factor associated with a higher likelihood of atopic dermatitis (AD) in our study. This finding contrasts with previous research suggesting that a greener environment is typically protective against allergies, particularly allergic asthma and allergic rhinitis [37]. This intriguing finding prompts us to consider the role of environmental factors in AD development [38]. Previous studies have proposed that the presence of a garden may introduce infants to a wider range of allergens which could influence their immune system’s response and increase the AD risk [39,40]. Additionally, the use of chemicals in the garden, such as insecticides and pesticides, as suggested by Buralli et al. in their review [41], could contribute to this phenomenon. Moreover, residing in a house with a garden may be associated with heightened hygiene measures, such as frequent hand-washing and longer bathing, which can contribute to skin dryness and trigger eczema flare-ups [42,43]. Nevertheless, further research is imperative to establish clear and definitive conclusions regarding the intricate relationship between home gardens, environmental factors, and AD development.

The study is subject to certain limitations, such as potential recall bias in parental reporting. Furthermore, information about the infant’s birth and upbringing environment was restricted to the presence of a garden. We did not explore whether gardens were maintained with possible irritants and proinflammatory elements (e.g., pesticides or herbicides), or consider factors like air pollution and the overall greenness in the environment.

## 5. Conclusions

The study emphasizes the complexity of AD and food allergies in infants with AD. Mothers of infants with atopy should refrain from smoking during future pregnancies, strive for vaginal delivery, and encourage a diverse diet upon introducing complementary foods. This diet should be rich in plant-based foods while avoiding the late introduction of common food allergens without a valid reason. Future studies should investigate the impact of green environments and related environmental factors on the development of AD and food allergies.

## Figures and Tables

**Table 1:** Demographic characteristics and personal atopy history of the study participants.

	Total Cohort (n = 501)	AD (n = 87)	Non-AD (n = 414)	*p*-Value
Age, years	2 (2–3)	2 (2–3)	2 (2–3)	0.50
Sex, male	268 (54%)	49 (56%)	219 (53%)	0.64
**Place of birth**				0.04
Thessaloniki	165 (60%)	15 (43%)	150 (62%)	
Crete	112 (40%)	20 (57%)	92 (38%)	
Nationality, Greek	474 (92%)	81 (93%)	390 (94%)	0.80
Age at diagnosis (months)		5.5 (2–7)	n/a	n/a
Weight at diagnosis (kg)		12.3 (6.8–13.4)	n/a	n/a
Height at diagnosis (cm)		69 (68–70)		n/a
Food elimination by doctor (because of AD)		9 (10%) (1 egg, 4 milk, 2 nuts, 2 other)		
**Food elimination without doctors’ guidance**	45 (9%)	33 (37.9%)	12 (2.9%)	0.004
**Reason for elimination**				
Child refuses to try	13 (2.59%)	13 (14.9%)	4 (0.97%)	
Mom considers it as highly allergenic	15 (2.99%)	11 (12.64%)	4 (0.97%	
Mom does not consider it as healthy choice	9 (1.8%)	9 (10.34%)	4 (0.97%)	
Mom afraid of AD exacerbation	8 (1.6%)	8 (9.2%)	0	
**Main Foods eliminated**				
Milk	5 (1%)	5 (5.75%)	0	
Egg	2 (0.4%)	2 (2.30%)	0	
Peanut	2 (0.4%)	1 (2.3%)	2 (0.48%)	
Sesame	1 (0.2%)	0 (0%)	2 (0.48%)	
Lentils	1 (0.2%)	1 (1.15%)	0	
Pulses (beans, chickpeas)	1 (0.2%)	1 (1.15%)	0	
Shellfish	2 (0.4%)	2 (9.2%)	0	
Strawberry	4 (0.2%)	4 (1.15%)	0	
Fig	3 (0.6%)	3 (3.45%)	0	
Orange	2 (0.4%)	2 (9.2%)	0	
Peach	3 (0.6%)	3 (3.45%)	0	
Apple	2 (0.4%)	2 (9.2%)	0	
Kiwi	1 (0.2%)	1 (1.15%)	0	
Beef	3 (0.6%)	3 (3.45%)	0	
Spicy foods	1 (0.2%)	1 (1.15%)	0	
Sugar	4 (0.8%)	4 (4.60%)	0	
**Food Allergy**				0.008
No	431 (86%)	64 (74%)	367 (88%)	
Milk	40 (8%)	12 (14%)	28 (7%)	
Egg	6 (1%)	3 (3%)	3 (1%)	
Nuts	4 (1%)	1 (1%)	3 (1%)	
Other	21 (4%)	7 (8%)	14 (3%)	
Anaphylaxis	6 (1%)	3 (3%)	3 (1%)	0.07
**Food that caused anaphylaxis**				1.00
Milk	4 (31%)	2 (40%)	2 (25%)	
Egg	8 (61%)	3 (60%)	5 (63%)	
Sesame/Tahini	1 (8%)	0	1 (12%)	
Emergency Kit	6 (1%)	5 (8%)	1 (0.2%)	0.001

AD: atopic dermatitis; kg: kilograms; cm: centimeters; n/a (not applicable). Values are presented as mean (±SD), median (IQR), or sum (percentage). Statistically significant differences were considered *p*-values ≤ 0.05.

**Table 2:** Familial characteristics and atopic history.

	Total Cohort (n = 501)	AD (n = 87)	Non-AD (n = 414)	*p*-Value
**Maternal Education**				1.00
Elementary	2 (0.5%)	0	2 (0.5%)	
High School	167 (34%)	29 (34%)	138 (34%)	
Technical School	79 (16%)	14 (17%)	65 (16%)	
University	241 (49.5%)	41 (49%)	200 (49.5%)	
**Paternal Education**				0.10
Elementary	9 (2%)	4 (5%)	5 (1%)	
High School	200 (42%)	38 (46%)	162 (42%)	
Technical School	73 (16%)	13 (16%)	60 (16%)	
University	188 (40%)	27 (33%)	161 (42%)	
**Maternal atopy history**				0.05
No	383 (79%)	62 (75%)	320 (79%)	
Food allergy	39 (8%)	4 (5%)	35 (9%)	
Asthma	16 (3%)	6 (7%)	10 (3%)	
Eczema	9 (2%)	4 (5%)	5 (1%)	
AD	6 (1%)	2 (2%)	4 (1%)	
Rhinitis	30 (6%)	5 (6%)	25 (6%)	
Drugs	4 (1%)	0	4 (1%)	
**Paternal Atopy history**				0.02
No	376 (77%)	62 (72%)	314 (78%)	
Food allergy	45 (9%)	4 (5%)	41 (10%)	
Asthma	21 (4%)	4 (5%)	17 (4.2%)	
Eczema	9 (2%)	2 (2%)	7 (2%)	
AD	2 (0.4%)	1 (1%)	1 (0.2%)	
Rhinitis	26 (5%)	11 (13%)	15 (4%)	
Drugs	12 (2%)	2 (2%)	10 (3%)	
Maternal smoking in pregnancy	63 (13%)	20 (23%)	43 (10%)	0.002
Maternal smoking in breastfeeding	48 (10%)	15 (17%)	33 (9%)	0.02
Maternal smoking during the first 3 years of life	122 (25%)	25 (30%)	97 (24%)	0.27
Paternal smoking during the first 3 years of life	265 (53%)	46 (53%)	219 (53%)	1.00
Number of siblings	1 (0–1)	1 (0–1)	1 (0–1)	0.54
Number of older siblings	0 (0–1)	1 (0–1)	0 (0–1)	0.04
**Older Sibling atopic history**				0.005
No	361 (91%)	45 (78%)	316 (93%)	
Food allergy	12 (3%)	5 (9%)	7 (2%)	
Asthma	7 (2%)	2 (3%)	5 (2%)	
Eczema	4 (1%)	2 (3%)	2 (1%)	
AD	4 (1%)	1 (2%)	3 (1%)	
Rhinitis	9 (2%)	3 (5%)	6 (2%)	
Drugs	1 (0.3%)	0	1 (0.3%)	

AD: atopic dermatitis. Values are presented as sum (percentage). Statistically significant differences were considered *p*-values ≤ 0.05.

**Table 3:** Perinatal factors of the studied population.

	Total Cohort (n = 501)	AD (n = 87)	Non-AD (n = 414)	*p*-Value
Gestational age, weeks	34.4 ± 2.8	34.6 ± 3.4	34.3 ± 2.8	0.73
Birth weight, gr	3073 ± 629	3079 ± 657	3071 ± 626	0.92
**Mode of Delivery**				0.006
Vaginal	166 (33%)	40 (47%)	126 (31%)	
Cesarean section	333(67%)	46 (53%)	287 (69%)	
Duration of breastfeeding, months	4 (0.3–12)	3 (1.1–14.5)	4 (0–12)	0.07
Duration of exclusive breastfeeding, months	3 (0–6)	2 (0–5)	3 (0–6)	0.10
**Food elimination during pregnancy**	99 (22%)	17 (22%)	82 (23%)	1.00
Milk	4 (7%)	0	4 (10%)	0.65
Beef	9 (175)	3 (25%)	6 (14%)	
Fish	9 (17%)	3 (35%)	6 (14%)	
Bread	4 (7%)	1 (8%)	3 (7%)	
Spicy food beans	5 (9%)	0	5 (12%)	
Other	23 (43%)	5 (42%)	18 (43%)	
**Reason for elimination in pregnancy**				0.40
Never had it in her diet	12 (15%)	3 (20%)	9 (14%)	
Does not consider it a healthy choice	22 (27%)	4 (27%)	18 (28%)	
GI symptoms when consumed	26 (33%)	2 (13%)	24 (36%)	
Fear of increasing the infant’s allergy risk	13 (16%)	4 (27%)	9 (14%)	
Other	7 (9%)	2 (13%)	5 (8%)	
**Food elimination during breastfeeding**				0.001
Milk	20 (4%)	4 (5%)	16 (4%)	
Nuts	14 (3%)	5 (6%)	9 (2%)	
Pulses	9 (2%)	3 (4%)	6 (2%)	
Other	35 (7%)	14 (17%)	21 (5%)	
**Reason for elimination in breastfeeding**				<0.001
Never had it in her diet	9 (3%)	2 (4%)	7 (3%)	
Does not consider it a healthy choice	13 (4%)	2 (4%)	11 (4%)	
GI symptoms when consumed	16 (5%)	7 (15%)	9 (4%)	
Fear of inducing the infant’s AD exacerbation	20 (7%)	6 (13%)	14 (6%)	
Observed symptoms in the infant	15 (5%)	7 (15%)	8 (3%)	
Antibiotics during pregnancy	78 (16%)	12 (14%)	66 (16%)	0.63
Area of living				0.88
Urban	397 (80%)	69 (80%)	328 (79%)	
Rural	103 (20%)	17 (20%)	86 (21%)	
House with a garden	187 (38%)	44 (51%)	143 (35%)	0.005
Pets	158 (32%)	25 (29%)	133 (33%)	0.53
Type of pet				1.00
Dog	107 (82%)	19 (90%)	88 (81%)	
Cat	10 (7%)	1 (5%)	9 (8%)	
Horse	1 (1%)	0	1 (1%)	
Chicken	2 (2%)	0	2 (2%)	
Canary	2 (2%)	0	2 (2%)	
Fish/Turtle	2 (2%)	0	2 (2%)	
Other	5 (4%)	1 (5%)	4 (4%)	
Age that a pet was introduced in the household (months)	1 (0.1–4)	1 (1–7)	1 (0–4)	0.54

AD: atopic dermatitis; GI: gastrointestinal. Values are presented as mean (±SD), median (IQR), or sum (percentage). Statistically significant differences were considered *p*-values ≤ 0.05.

**Table 4:** Complementary feeding and early life environmental factors.

	Total Cohort (n = 501)	AD (n = 87)	Non-AD (n = 414)	*p*-Value
Formula feeding, months	3 (1–6)	3 (1–6)	3 (1–6)	0.87
Formula type				0.66
Normal	249 (73%)	38 (83%)	211 (72%)	
Partial hydrolyzed	58 (17%)	6 (13%)	52 (18%)	
Fully hydrolyzed	9 (3%)	0	9 (3%)	
Elemental	15 (4%)	1 (2%)	14 (5%)	
Other	7 (3%)	1 (2%)	6 (2%)	
Complementary feeding, months	6 (5–6)	6 (5–6)	6 (5–6)	0.76
Foods in pieces, months	9 (7–11)	8 (6–11)	9 (7–11)	0.19
Vegetables, months	6 (5.5–6)	6 (5.2–6)	6 (5.5–6)	0.47
Vegetables delayed after 12 months	10 (0–14)	4 (0–10)	10 (3–14)	0.002
Fruits, months	6 (5–6)	6 (5–6)	6 (5–6)	0.36
Fruits delayed after 12 months	10 (5–10.4)	10 (3–10)	10 (5–10.4)	0.02
Starch–gluten, months	8 (6–11)	8 (7–10)	8 (6–12)	0.41
Trahanas, months	10 (7–12)	9.5 (7–11)	10 (7–12)	0.33
Oat, months	8 (7–12)	8 (7–10)	9 (7–12)	0.21
Fish, months	11 (8–12)	10 (8–12)	11 (8–12)	0.52
Hard-boiled egg, months	10 (8–12)	9 (8–12)	10 (8–12)	0.66
Runny egg, months	10 (8–12)	10 (8–12)	10 (8–12)	0.97
Raw egg, months	1 (1–1)	1 (1–1)	1 (1–1)	0.30
Nuts, months	13 (12–18)	12 (11–14)	14 (12–20)	0.02
Nut butter, months	12 (11–20)	12 (10–17)	13 (11–20)	0.33
Peanut, months	15 (12–24)	13 (12–24)	16 (12–24)	0.77
Peanut butter, months	13 (12–24)	12 (12–18)	13 (12–24)	0.93
Sesame, months	12 (10–12)	12 (10–12.5)	12 (10–12)	0.48
Tahini, months	12 (11–16)	12 (12–14)	12 (11–16)	0.54
Vegetables first included				0.19
Not yet started	42 (94%)	2 (67%)	40 (96%)	
Carrot	1 (2%)	0	1 (2%)	
Sweet potato	1 (2%)	1 (33%)	0	
Tomato	1 (2%)	0	1 (2%)	
Type of fish				0.56
Cod	138 (54%)	34 (62%)	104 (52%)	
Redfish	38 (15%)	8 (15%)	30 (15%)	
Bream	37 (15%)	5 (9%)	32 (16%)	
Perch	4 (2%)	1 (2%)	3 (2%)	
Sole	13 (5%)	2 (4%)	11 (6%)	
Pike	1 (0.4%)	0	1 (1%)	
Bass	3 (1%)	0	3 (2%)	
Salmon	1 (0.4%)	0	1 (2%)	
Fish	1 (0.4%)	1 (2%)	0	
Scorpion	4 (2%)	2 (4%)	2 (1%)	
Swordfish	1 (0.4%)	1 (2%)	0	
Pagasius	1 (0.4%)	0	1 (1%)	
Combination	13 (5%)	1 (2%)	12 (8%)	
Antibiotics times	0 (0–1)	0 (0–1)	0 (0–1)	0.12
Antibiotics first use, months	4 (1–12)	8 (5–12)	6 (2–12)	0.54
Antibiotics during the first 3 years of age	204 (41%)	32 (37%)	172 (42%)	0.47

AD: atopic dermatitis. Values are presented as mean (±SD), median (IQR), or sum (percentage). Statistically significant differences were considered *p*-values ≤ 0.05.

**Table 5:** Logistic regression analysis in infants and the risk of AD.

	OR	95% CI	*p*-Value
**AD (Univariate analysis)**			
Crete	2.17	1.06–4.54	0.03
Mode of delivery, vaginal delivery	1.19	1.23–3.17	0.005
House with garden	1.97	1.23–3.16	0.004
Maternal smoking in pregnancy	2.56	1.42–4.63	0.002
Paternal atopy history	1.18	1.03–1.35	0.01
Older sibling atopy history	1.33	1.07–1.66	0.01
**AD (Multivariate analysis)**			
Mode of delivery, vaginal delivery	3.11	1.27–7.61	0.006
House with garden	3.84	1.50–9.80	0.01
Maternal smoking in pregnancy	7.18	2.27–22.64	0.001
Older sibling atopy history	7.87	1.25–50.00	0.03

AD: atopic dermatitis; OR: odds ratio; 95% CI: 95% confidence interval. Statistically significant differences were considered *p*-values ≤ 0.05.

**Table 6:** Risk factor of food allergy in infants with AD.

	OR	95% CI	*p*-Value
**Food allergy (Univariate analysis)**			
Unsupervised food elimination before the onset of food allergy, yes	6.11	1.30–28.53	0.02
Mode of delivery, cesarean section	3.44	1.10–10.75	0.03
**Food allergy (Multivariate analysis)**			
Unsupervised food elimination before the onset of food allergy, yes	6.63	1.31–33.46	0.02
Mode of delivery, cesarean section	3.58	1.05–12.19	0.04

AD: atopic dermatitis; OR: odds ratio; 95% CI: 95% confidence interval. Statistically significant differences were considered *p*-values ≤ 0.05.

## Data Availability

The data that support the findings of this study are available on request from the corresponding author.
